# Clinical Risk Score for Invasive Pulmonary Aspergillosis in Patients With Liver Failure: A Retrospective Study in Zhejiang

**DOI:** 10.3389/fmed.2021.762504

**Published:** 2021-11-22

**Authors:** Xuan Zhang, Sijia Shen, Xiahong Dai, Yunjiao Bi, Junjie Zhang, Yuhao Wu, Yishang Shi, Runan Wei, Hainv Gao

**Affiliations:** ^1^State Key Laboratory for Diagnosis and Treatment of Infectious Diseases, National Clinical Research Center for Infectious Diseases, National Medical Center for Infectious Diseases, Collaborative Innovation Center for Diagnosis and Treatment of Infectious Diseases, The First Affiliated Hospital, College of Medicine, Zhejiang University, Hangzhou, China; ^2^College of Medicine, Zhejiang University, Hangzhou, China; ^3^Shulan (Hangzhou) Hospital Affiliated to Zhejiang Shuren University Shulan International Medical College, Hangzhou, China; ^4^Shulan International Medical College, Zhejiang Shuren University, Hangzhou, China

**Keywords:** liver failure, invasive pulmonary aspergillosis, prophylaxis, voriconazole, risk score

## Abstract

**Purpose:** The mortality of invasive pulmonary aspergillosis (IPA) in patients with liver failure was high. However, the prophylactic treatment in those patients with a high-risk factor in IPA has not been researched.

**Patients and methods:** A multicenter, retrospective study was conducted in patients with liver failure. The study cohort of liver failure was randomly split into a training set for model development and the other served as the testing set for model verification. Multivariate analysis was performed to identify the risk factors of IPA. A weighted risk score for IPA was established. Anti-fungal treatment was prophylactically used in patients with medium and high IPA risk to evaluate the effect.

**Results:** In total, 1,722 patients with liver failure were enrolled. Fifty-seven patients who received prophylactic treatment were excluded from the risk factor system study. About 1,665 patients were randomly split at a ratio of 2:1 into two datasets. Diabetes, glucocorticoids, plasma exchange, and hepatorenal syndrome (HRS) were risk factors in IPA in patients with liver failure, with weighted risk scores of 4, 7, 2, and 3, respectively. In the validation set and test set, the patients with risk scores of ≤ 3 presented low incidences of IPA at 4 and 2.7%. Patients with risk scores of 4–5 had an IPA incidence of 7.6% and 10.1%, and could be considered as a medium-risk group (*p* < 0.01 vs. the group with scores of ≤ 3), whereas those with risk scores of >5 manifested a significantly higher IPA incidence of 21.2 and 12.7%, who were considered a high-risk group (*p* < 0.01 vs. the groups with scores of 4–5 and >5, respectively). The IPA risk scores in the training set and the testing set were also analyzed by the ROC with an area under the ROC of 0.7152 and 0.6912. In this study, 57 patients received antifungal prophylaxis; the incidence of IPA was 1.8%, which was significantly lower after prophylactic antifungal therapy (*p* < 0.001).

**Conclusions:** A weighted risk score for patients with liver failure, complicated with IPA, was established and confirmed in the testing cohort. Voriconazole prophylactic treatment to patients with liver failure with medium and high IPA risk can effectively prevent Aspergillus infection.

## Introduction

Invasive pulmonary aspergillosis (IPA) is a devastating infectious disease in patients with liver failure; the mortality rate exceeds 80% (1, 2). Early diagnosis and treatment of IPA are particularly important. However, clinical diagnosis of IPA in liver failure is a huge challenge. The traditional G test and the GM test can diagnose IPA early in patients with allogeneic hematopoietic stem cell transplantation, but they lack sensitivity in patients with liver failure (3). Invasive diagnostic procedures are often not feasible due to prolonged prothrombin time and thrombocytopenia. So, at present, the clinical diagnosis of liver failure complicated with IPA infection depends more on symptom-triggered pulmonary CT screening. However, due to the lack of specificity in clinical symptoms and imaging of liver failure complicated with IPA infection, the study found that the positive predictive value of this method was 61% and the negative predictive value was 92% (4). Moreover, previous studies have shown that patients with IPA were diagnosed when the CLIF-SOFA lung score is >1 and had the worst prognosis, poor antifungal treatment effect, and the highest mortality (5). These studies demonstrated that early diagnosis and early treatment are the key factors to improve the prognosis of patients with liver failure, complicated with IPA infection, but it was difficult to achieve.

In view of the disastrous consequences of IPA in patients with liver failure, the risk factor in IPA has been researched extensively, such as hemodialysis and prior antibiotics use (3), prolonged and high-dose corticosteroid therapy (6, 7), and recent history of neutropenia (8, 9). The prophylactic antifungal treatment, which was based on the risk factors, had been evaluated in multiple clinical trials in different diseases. Rijnders et al. showed that prophylactic inhalation of liposomal amphotericin B significantly reduced the incidence of IPA in patients during prolonged neutropenia (10). The China Assessment of Antifungal Therapy in Hematological Diseases (CAESAR) study showed antifungal prophylaxis was beneficial in patients with hematological malignancies with an intermediate and high risk of invasive fungal disease (11). A clinical trial at Mayo Clinic (12) researched lung transplant recipients who receive prolonged and mostly lifelong azole antifungal prophylaxis; none of the patients developed disseminated invasive aspergillosis. However, the prophylactic treatment in patients with liver failure with a risk factor for IPA has not been researched.

In this study, we established a weighted risk score for IPA that accurately discriminated a cohort of patients with liver failure with low, intermediate, and high risks of IPA. Then, the efficacy of antifungal prophylaxis with voriconazole or caspofungin was evaluated in patients with different IPA risks in order to reduce mortality in this population.

## Materials and Methods

### Study Design

A multicenter, retrospective observational study was carried out in patients with liver failure admitted to two tertiary hospitals in Zhejiang province from December 2008 to July 2021: one is Shulan Hangzhou Hospital; the other is the First Affiliated Hospital, School of Medicine, Zhejiang University. The collected data included baseline characteristics, type of liver failure, clinical features, underlying disease, complication, antifungal treatment, treatment-related potential risk factors of IPA, and the prognosis. Because the study was retrospective and the data were analyzed anonymously, the need for consent was waived.

### Enrollment Criteria

Liver failure was defined according to the Diagnosis and Treatment Guideline for Liver Failure in China (2018) (13). The definition of IPA was used by the European Organization for Research and Treatment of Cancer (EORTC) consensus (14), which was diagnosed if the following two criteria were fulfilled: (1) positive culture of Aspergillus spp. from sputum; (2) presence of one of the following three signs on computed tomography: dense, well-circumscribed lesions with or without a halo sign, air-crescent sign, or cavity.

### Statistical Analysis

The study cohort of liver failure was randomly split at a ratio of 2:1 into two sets, of which one served as the training set for model development and the other served as the testing set for model verification. The characteristics of patients were compared between two datasets using Student's *t*-tests or Kruskal-Wallis tests for continuous variables where appropriate and χ^2^ or Fisher exact tests for categorical variables. We first, by using univariate analysis with *p* < 0.10, identified the risk factors that were individually complicated with probable IPA. The factors that demonstrated an individual association were demonstrated the multivariate logistic regression with the stepwise criteria of 0.05. Points were assigned for the variables and were weighted approximately by the corresponding regression β-coefficients. Receiver operator curves (ROC) were calculated to assess the discrimination capacity of the risk score. Once the model was determined, it was tested in the test set to confirm its performance in predicting the IPA incidence. All statistical analyses were performed using SPSS version 18.0 (SPSS, Chicago, IL, USA).

## Results

### Study Populations and Incidence of IPA

In this study, 1,722 patients with liver failure were enrolled. Fifty-seven patients who received voriconazole or caspofungin prophylactic treatment were excluded from the risk factor system study. Of the remaining 1,665 patients, the patients with IPA were 111, the overall incidence of IPA was 6.7%, and the mortality of IPA was 85.6%. Acute (subacute) liver failure, acute-on-chronic (subacute-on-chronic) liver failure, and chronic liver failure were 198, 902, and 565 cases, and the IPA incidences were 7.1, 7.1, and 5.9%, respectively. There was no significant difference between any two groups for IPA incidence.

About 1,665 liver failure patients were randomly split at a ratio of 2:1 into two datasets, 1,155 patients were enrolled in the training set, and 510 patients were in the testing set.

The characteristics of patients in training and testing datasets were similar in all aspects, as shown in Table 1.

**Table 1:** Characteristics of patients with liver failure in the training and testing sets.

**Characteristic**			**Training set**	**Testing set**	***P* value**
			**(*n* = 1,155)**	**(*n* = 510)**	
Sex	Male		887 (76.8%)	391 (76.7%)	0.9499
	Female		268 (23.2%)	119 (22.3%)	
Age	Mean (S.D.)		49.96 (13.61)	50.63 (13.22)	0.2606
Diagnosis	ALF^a^		98 (8.5%)	44 (8.6%)	0.0604
	SALF^b^		35 (3.0%)	20 (3.9%)	
	ACLF^c^		649 (56.2%)	252 (49.4%)	
	CLF^d^		370 (32.0%)	193 (37.8%)	
Etieology	Hepatitis B		774 (67.0%)	323 (63.3%)	0.4003
	Hepatitis E		9 (0.8%)	6 (1.2%)	
	Alcohol		67 (5.8%)	34 (6.7%)	
	Drug		77 (6.7%)	30 (5.9%)	
	Autoimmunity		27 (2.3%)	15 (2.9%)	
	Hepatolenticular degeneration		8 (0.7%)	2 (0.4%)	
	Schistosome		7 (0.6%)	4 (0.8%)	
	Cryptogenic		58 (5.0%)	39 (7.6%)	
	Malignancy		52 (4.5%)	22 (4.3%)	
	Two or more factors		53 (4.6%)	27 (5.3%)	
	Other		19 (1.6%)	5 (1.0%)	
underlaying diseases	Diabetes	Yes	119 (10.3%)	56 (11.0%)	0.7423
		No	1,036 (89.7%)	454 (89.0%)	
	Malignancy	Yes	151 (13.1%)	63 (13.3%)	0.7447
		No	1,004 (86.9%)	447 (87.6%)	
Complications	Neutropenia	Yes	22 (1.9%)	14 (2.7%)	0.3660
		No	1,133 (98.1%)	496 (97.3%)	
	Gastrointestinal bleeding	Yes	160 (13.9%)	84 (16.5%)	0.1878
		No	995 (86.1%)	426 (83.5%)	
	HE^e^	Yes	504 (43.6%)	221 (43.3%)	0.9511
		No	651 (56.4%)	289 (56.7%)	
	HRS^f^	Yes	187 (16.2%)	85 (16.7%)	0.8647
		No	968 (83.8%)	425 (83.3%)	
Liver function	TBil^g^	Median	419.00	436.00	0.1583
		Min, Max	170.00, 3,015.00	171.00,990.00	
	INR^h^	Median	2.58	2.64	0.8341
		Min, Max	1.50,12.30	1.51,6.52	
Treatment	Antibiotic usage^i^	Yes	822 (71.2%)	372 (72.9%)	0.4958
		No	333 (28.8%)	138 (27.1%)	
	Steroid exposure^j^	Yes	88 (7.6%)	35 (6.9%)	0.6583
		No	1,067 (92.4%)	475 (93.1%)	
	Plasma exchange	Yes	631 (54.6%)	274 (53.7%)	0.7726
		No	524 (45.4%)	236 (46.3%)	
Prognosis	Liver transplantion		196 (17.0%)	89 (17.5%)	0.8617
	Recovery		318 (27.5%)	139 (27.3%)	
	Demise		220 (19.0%)	107 (21.0%)	
	Transfer to another hospital		58 (5.0%)	26 (5.1%)	
	Give up treatment		363 (31.4%)	149 (29.2%)	
IPA		Yes	81 (7.0%)	30 (5.9%)	0.6222
		No	1,074 (92.9%)	480 (94.1%)	

a *ALF, acute liver failure*;

b *SALF, subacute liver failure*;

c *ACLF, acute-on-chronic liver failure*;

d *CLF, chronic liver failure*;

e *HE, hepatic encephalopathy*;

f *HRS, hepatorenal syndrome*;

g *TBil, total bilirubin*;

h *INR, international normalized ratio*;

i *antibiotic usage, antimicrobial agent use for ≥5 days*;

j *steroid exposure, steroid treatment for ≥7 days, maximum dosage (equivalent methylprednisolone) ≥40 mg/day*.

### Risk Scores Associated With IPA in the Training Dataset

The variables of the training set that was associated with IPA incidence, including diabetes, corticosteroid, plasma exchange treatment, and INR, were significant in the multivariate logistic regression, and weighted points were assigned (Table 2).

**Table 2:** Multivariate logistic regression analysis of risk factors associated with invasive pulmonary aspergillosis (IPA) development in the training dataset.

**Variable**	**Univariate logistic regression**				**Multivariate logistic regression**				
	**Coefficients**	**Standard error**	**Walds**	***p*-value**	**Coefficients**	**Weight of score**	**Standard error**	**Walds**	***p*-value**
Gender	0.3531	0.3031	1.165	0.244					
Age category	0.2551	0.2329	1.095	0.273					
Diabetes	0.5945	0.3203	1.856	0.0634	0.8256	4	0.3309	2.495	0.01258
Cancer	−0.4315	0.4061	1.063	0.288					
Antibiotic	0.4198	0.2815	1.491	0.136					
Corticosteroid	1.4576	0.2897	5.032	*p* < 0.01	1.5561	7	0.2974	5.232	p <0.01
Granulocytopenia	−0.5996	1.027	0.584	0.559					
Artifital liver treatment	0.4629	0.2423	1.91	0.0561	0.4286	2	0.249	1.722	0.03513
Gastrointestinal bleeding	0.3733	0.3000	1.245	0.213					
HRS	0.635	0.3576	3.123	0.10176					
HE	0.06876	0.23377	0.294	0.769					
Tbil	0.6228	0.3405	3.589	0.1012					
INR	0.7214	0.2442	2.954	0.00314	0.7128	3	0.2504	2.847	0.00441
Etiology	0.2851	0.2361	1.208	0.227					

*HRS, hepatorenal syndrome; HE, hepatic encephalopathy; Tbil (≥ 430 vs. <430) , total bilirubin; INR (≥ 2.6 vs. <2.6), international normalized ratio, etiology (HBV vs. others)*.

Based on the multivariate logistic regression analysis, IPA risk scores ranged from 0 to 16 (Table 3) and were calculated in the training set. The distribution of the risk scores and the cumulative incidence of IPA in liver failure patients were shown in Table 4. The patients with risk scores of ≤ 3 presented low incidences of IPA at 4%. Patients with risk scores of 4–5 had an IPA incidence of 7% and could be considered as a medium-risk group (*p* < 0.01 vs. the group with scores of ≤ 3), whereas those with risk scores of >5 manifested a significantly higher IPA incidence of 21.2%, who were considered as a high-risk group (*p* < 0.01 vs. the groups with scores of 4–5 and >5, respectively).

**Table 3:** Risk scores for IPA.

**Factor**	**Variables**	**Weight of score**
Diabetes	No	0
	YES	4
Corticosteroid	No	0
	YES	7
Plasma exchange	No	0
	YES	2
INR	No	0
	YES	3

**Table 4:** Distribution of risk scores vs. the cumulative incidence of IPA in the training and testing datasets.

**Risk scores**	**Patients (*n*)**	**IPA episodes (*n*)**	**IPA incidence (%)**
Training set			
≤ 3	649	26	4.0%
4~5^*^	330	25	7.6%
>5^**^	137	29	21.2%
Testing set			
≤ 3	296	8	2.7%
4–5^#^	139	14	10.1%
>5^##^	71	9	12.7%

* *p < 0.01 vs. the group with scores of ≤ 3*;

** *p < 0.01 vs. the groups with scores of 4–5 and >5, respectively*;

# *p < 0.01 vs. the group with scores of ≤ 3*;

## *p < 0.01 vs. the groups with scores of 4–5 and >5, respectively*.

**Figure 1:**
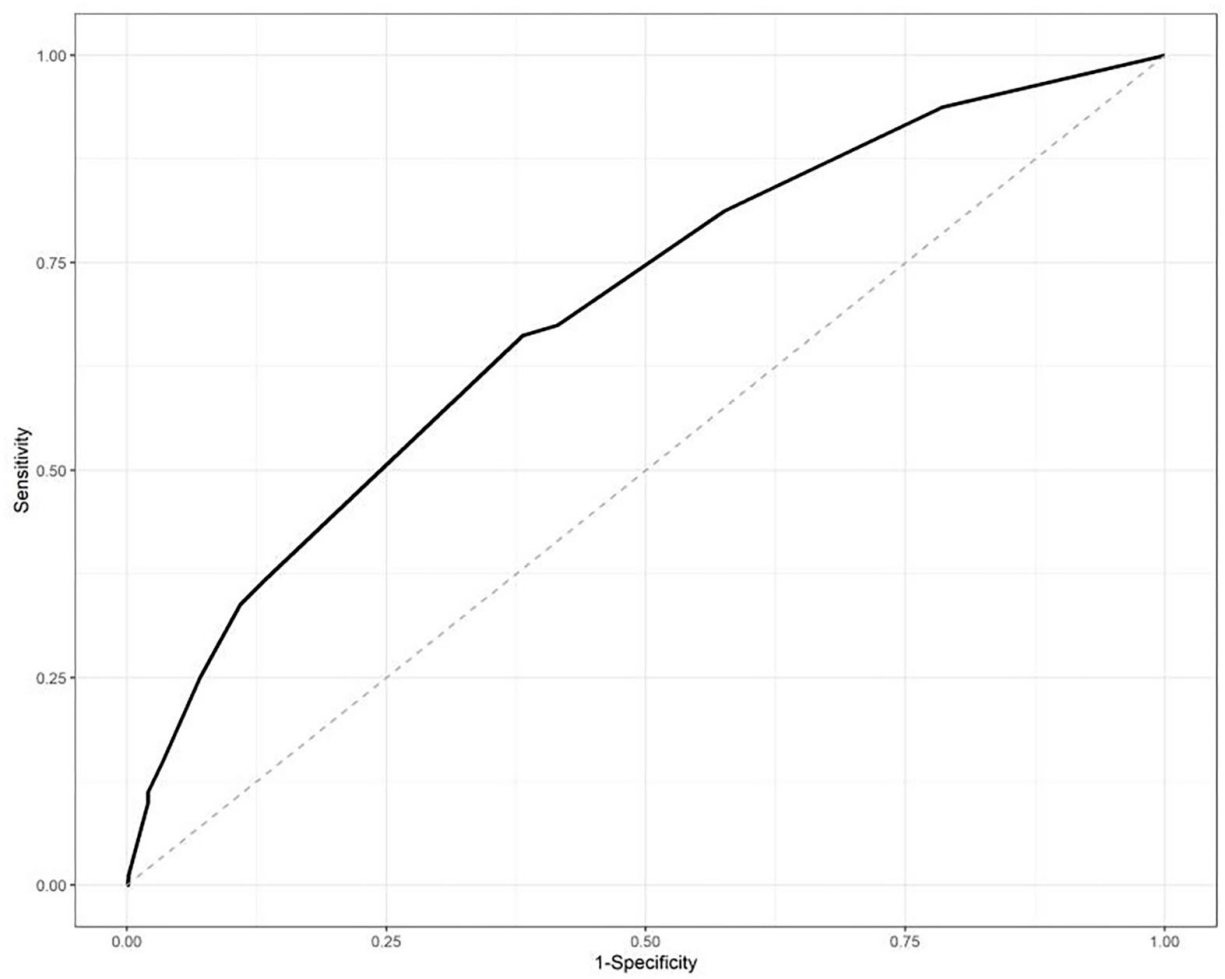
Receiver-operator curve (ROC) analysis of the risk scores in the training datasets. The area under the curve was 0.6912.

The ROC curve was used to identify the ability of the IPA risk scores, and the area under the curve was 0.6912, which is shown in Figure 1.

### Risk Scores Associated With IPA in the Testing Dataset

The IPA risk score was used to assess the patients with liver failure in the testing dataset. The incidence of IPA was 2.7% when the risk scores of IPA were less than or equal to 3. As the risk scores reached 4–5, the IPA incidence increased to 7% (*p* < 0.01 vs. the group with scores of ≤ 3), whereas the IPA incidence was 12.7% in the patients with risk scores of >5 (*p* < 0.01 vs. the groups with scores of 4–5 and >5, respectively).

**Figure 2:**
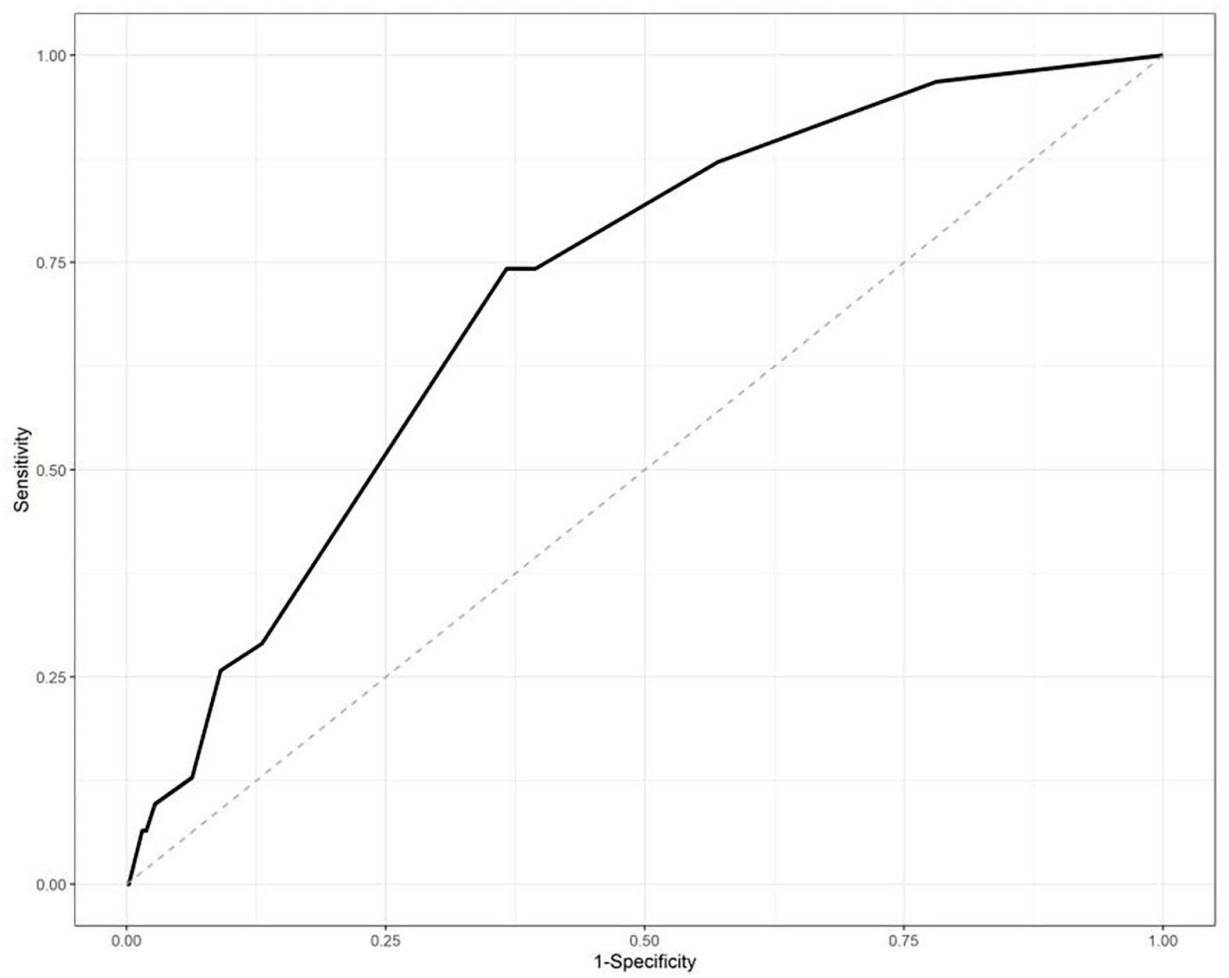
Receiver-operator curve (ROC) analysis of the risk scores in the testing datasets. The area under the curve was 0.7152.

The IPA risk scores in the testing set were also analyzed by the ROC with an area under the ROC of 0.7152, as shown in Figure 2.

### Impact of Antifungal Prophylaxis in Patients With Different Risk Scores

In this study, 57 patients received antifungal prophylaxis, who had significantly more seriously impaired liver function than patients without prophylaxis (*p* < 0.001). Meanwhile, the risk scores were significantly higher in the population with antifungal prophylaxis (*p* < 0.001) (Table 5). However, the incidence of IPA was 1.8%, which was significantly lower after prophylactic antifungal therapy (*p* < 0.001).

**Table 5:** The characteristics of patients with or without antifungal prophylaxis and impact of antifungal prophylaxis in patients with different risk scores.

**Characteristics**		**No prophylaxis**		**Prophylaxis**		***P* value**
		***n***	**%**	***n***	**%**	
Sex						
	Male	1,269	76.9%	45	78.9%	0.8357
	Female	382	23.1%	12	21.1%	
Age						
	≤ 50	845	51.2%	24	42.1%	0.2252
	>50	806	48.8%	33	57.9%	
Corticosteroid						
	No	1,531	92.7%	54	94.7%	0.7943
	Yes	120	7.3%	3	5.3%	
Diabetes						
	No	1,477	89.5%	49	86.0%	0.5335
	Yes	174	10.5%	8	14.0%	
Plasma exchange treatment						
	No	749	45.4%	27	47.4%	0.8704
	Yes	902	54.6%	30	52.6%	
INR^a^						
	<2.6	829	50.2%	14	24.6%	<0.001
	≥2.6	822	49.8%	43	75.4%	
Risk scores (means ± sd)		3.5 ± 2.8		4.2 ± 2.6		<0.001
and IPA incidence (%)	≤ 3	945	3.6%	27	3.7%	
	4–5	469	11.4%	22	0	
	>5	237	18.3%	8	0	
IPA^b^						
	No	1,540	93.3%	56	98.2%	<0.001
	Yes	111	6.7%	1	1.8%	

a *INR, international normalized ratio*;

b *IPA, invasive pulmonary aspergillosis*.

## Discussion

Patients with end-stage liver diseases are now considered additional risk factors of IPA, along with allogeneic bone marrow transplantation (15). The prevalence of IPA in HBV-related ACLF patients has been reported to be 5–8.3%. The short-term mortality observed in these patients ranged from 73.5 to 100% (1, 5, 7). In our study, the incidence of IPA was 6.7%, and the mortality was 85.6%, which was consistent with previous reports. It is not difficult to find that the IPA rate in patients with liver failure is not high, but, once it occurs, the mortality is very high. Hence, numerous studies have reported lots of IPA risk factors of liver failure (1, 2, 7, 16, 17) in order to provide a way for clinical early diagnosis and early treatment.

The previous study showed antibiotic use was an independent factor associated with the occurrence of IPA in patients with liver failure (2), and the patients were prone to many types of infections caused by opportunistic pathogens, including Aspergillus spp. after antibiotics use. However, our results showed there was no significant difference in antibiotic use between the patients with IPA and those without. Instead, diabetes, INR, plasma exchange, and steroid use were proved to be significant risk factors for IPA. Diabetes and steroid use indirectly reflect the immune status, which was easier to understand that they are the risk factors of IPA in patients with liver failure. But, in this study, we, for the first time, confirmed that the plasma exchange therapy was the risk factor of IPA in liver failure; this risk score is 2, which belongs to the low-level risk factor. This means that patients with severe liver failure treated with plasma exchange received steroid treatment or had diabetes at the same time, which will increase the risk of IPA.

Over the past three decades, plasma exchange has been employed to treat liver failure. Due to the lack of randomized controlled studies, the effect of plasma exchange on liver failure has always been controversial (18). Until Larsen et al. (19) published the first randomized control trial of plasma exchange in patients with acute liver failure in 2016, then, plasmapheresis was added to the European guidelines (20) as Level I, Grade 1 recommendation in management of acute liver failure. Its proposed mechanism is the removal of plasma cytokines and drivers of systemic inflammatory cascade by plasma exchange. But why does plasma exchange increase the risk of Aspergillus infection? We speculate whether plasma exchange removes some cytokines at the same time, which mediate the inflow of macrophage and might limit the degree of local tissue destruction of Aspergillus infection (21).

Despite so many studies on risk factors, there is no study that has considered an early prophylactic treatment for high-risk patients. There may be two reasons that hinder the measures to be taken for clinical prophylactic treatment. First, the risk factors found in different studies are inconsistent, there is no further verification of these risk factors, and there is no distinction between low-risk, medium-risk, and high-risk factors. So, it is difficult for clinical doctors to judge all of these risk factors and take steps. In fact, almost all liver failure will have 1–2 risk factors above mentioned, but it is not impossible to give prophylactic antifungal therapy to all the patients with liver failure; second, it is difficult to determine which prophylactic method has to be taken. If voriconazole prophylactic treatment is taken in line with HSCT (11), how much dosage of voriconazole needs to be used in patients with liver failure, because of the hepatotoxicity of voriconazole, whether its prophylactic use will be harmful to the liver of patients with liver failure. Different from creatinine clearance, the pharmacokinetics of drugs metabolized from the liver with different disease severities is not a simple linear relationship, and there are too many concerns about voriconazole metabolism and human CYP2C19 gene polymorphism at the same time (22, 23).

In this study, we conducted a multicenter-based study to build up a risk score system for IPA in patients with liver failure. We confirmed the discriminative performance of the IPA risk score system in both the training and testing sets. The effective concentration range of voriconazole is between 1 and 5 ug/ml. As long as the concentration is monitored, the risk can be avoided. Prevention failed in one patient among 27 low-risk patients, who were prevented with caspofungin. However, what we need to further study is the difference between caspofungin and voriconazole in the prevention of Aspergillus infection.

There are several limitations to our study. First, we can only, according to the results of sputum culture and pulmonary CT, diagnose IPA in patients with liver failure due to their coagulation function and platelet status, which will not lead to a precise diagnosis; second, this study is a retrospective analysis, which could lead to the unbalanced distribution of confounding factors when we evaluate the efficacy of the prophylactic treatment. Some factors may affect the results, such as the duration of the disease, the severity of the disease, or the dosage of the prophylactic antifungal drug. Third, we included liver failure caused by different causes, and they have different pathogenesis, which will affect the incidence rate of IPA. Finally, the sample size was insufficient to compare different prophylactic treatment effects in different subgroups.

## Conclusion

To the best of our knowledge, this is the first report for developing an IPA risk score system based on a large patient population with liver failure. We established a weighted risk score for IPA that could reliably discriminate the incidence of IPA. The precise risk assessment of IPA may provide a chance for risk based antifungal treatment in patients with liver failure. For the first time, we tried to give voriconazole prophylactic treatment to patients with liver failure with medium and high IPA risk and found that it can effectively prevent Aspergillus infection. Furthermore, we need to expand the sample size and design a multicenter prospective study to further verify the effect of preventive antifungal therapy in high-risk groups and explore the best dose of preventive therapy in patients with liver failure.

## Data Availability Statement

The original contributions presented in the study are included in the article/supplementary material, further inquiries can be directed to the corresponding author/s.

## Ethics Statement

The studies involving human participants were reviewed and approved by Shulan Hangzhou Hospital. Written informed consent from the participants' legal guardian/next of kin was not required to participate in this study in accordance with the national legislation and the institutional requirements.

## Author Contributions

HG designed and conceptualized the study. XZ, SS, and XD designed the table to collect and analyze the data. YB, YW, YS, and JZ provided clinical data. XZ, SS, and HG wrote the manuscript. SS helped to revise the manuscript. All the authors have read and approved the final manuscript.

## Funding

This work was supported by the Zhejiang Basic Public Welfare Research Program (No. LGF21H030012) and the Zhejiang Non-public Medical Specialty Program.

## Conflict of Interest

The authors declare that the research was conducted in the absence of any commercial or financial relationships that could be construed as a potential conflict of interest.

## Publisher's Note

All claims expressed in this article are solely those of the authors and do not necessarily represent those of their affiliated organizations, or those of the publisher, the editors and the reviewers. Any product that may be evaluated in this article, or claim that may be made by its manufacturer, is not guaranteed or endorsed by the publisher.
